# A preclinical platform for assessing antitumor effects and systemic toxicities of cancer drug targets

**DOI:** 10.1073/pnas.2110557119

**Published:** 2022-04-20

**Authors:** Xiang Li, Chun-Hao Huang, Francisco J. Sánchez-Rivera, Margaret C. Kennedy, Darjus F. Tschaharganeh, John P. Morris, Antonella Montinaro, Kevin P. O'Rourke, Ana Banito, John E. Wilkinson, Chi-Chao Chen, Yu-Jui Ho, Lukas E. Dow, Sha Tian, Wei Luan, Elisa de Stanchina, Tinghu Zhang, Nathanael S. Gray, Henning Walczak, Scott W. Lowe

**Affiliations:** ^a^Cancer Biology and Genetics Program, Memorial Sloan Kettering Cancer Center, Sloan Kettering Institute, New York, NY 10065;; ^b^Weill Cornell Graduate School of Medical Sciences, Cornell University, New York, NY 10021;; ^c^Centre for Cell Death, Cancer, and Inflammation, UCL Cancer Institute, University College London, London WC1E 6DD, United Kingdom;; ^d^Weill Cornell Medicine/The Rockefeller University/Sloan Kettering Institute Tri-Institutional MD-PhD Program, New York, NY 10065;; ^e^Department of Pathology, University of Michigan School of Medicine, Ann Arbor, MI 48109;; ^f^Innovative Medicines Accelerator, Stanford Chemistry, Engineering & Medicine for Human Health (ChEM-H), Stanford University, Stanford, CA 94305;; ^g^Cellular Stress Responses in Aging-Associated Diseases (CECAD), Cluster of Excellence, University of Cologne, Cologne 50931, Germany;; ^h^Center for Biochemistry, Medical Faculty, University of Cologne, 50931 Cologne, Germany;; ^i^Howard Hughes Medical Institute, Memorial Sloan Kettering Cancer Center, New York, NY 10065

**Keywords:** preclinical platform, CDK9, mouse model, hepatocellular carcinoma

## Abstract

Anticancer drug development campaigns often fail due to an incomplete understanding of the therapeutic index differentiating the efficacy of the agent against the cancer and its on-target toxicities to the host. To address this issue, we established a versatile preclinical platform in which genetically defined cancers are produced using somatic tissue engineering in transgenic mice harboring a doxycycline-inducible short hairpin RNA against the target of interest. In this system, target inhibition is achieved by the addition of doxycycline, enabling simultaneous assessment of efficacy and toxicity in the same animal. As proof of concept, we focused on CDK9—a cancer target whose clinical development has been hampered by compounds with poorly understood target specificity and unacceptable toxicities. We systematically compared phenotypes produced by genetic *Cdk9* inhibition to those achieved using a recently developed highly specific small molecule CDK9 inhibitor and found that both perturbations led to robust antitumor responses. Remarkably, nontoxic levels of CDK9 inhibition could achieve significant treatment efficacy, and dose-dependent toxicities produced by prolonged CDK9 suppression were largely reversible upon *Cdk9* restoration or drug withdrawal. Overall, these results establish a versatile in vivo target validation platform that can be employed for rapid triaging of therapeutic targets and lend support to efforts aimed at advancing CDK9 inhibitors for cancer therapy.

## Significance

Many new cancer drugs fail at the clinical stage owing to poor efficacy and/or excessive toxicity, though whether this reflects shortcomings of the target or the drug is often unclear. To gain earlier insights into factors that can influence the therapeutic index of target inhibition in vivo, we combine inducible RNA interference and somatic engineering technologies to produce a cost-effective platform that enables systemic and inducible suppression of candidate target in normal tissues and tumor cells in the same mouse. By comparing the consequences of genetic and pharmacological CDK9 inhibition, we establish the utility of this platform to predict factors influencing the therapeutic index. Additionally, our studies provide support, and some cautionary notes, for the clinical development of CDK9 inhibitors.

Drug development is a time-consuming and resource-demanding process that often fails. One of the biggest hurdles between preclinical drug discovery and obtaining a valid clinical drug candidate is the step of drug target validation, which entails certifying a reasonable therapeutic index according to differential on-target efficacy and the toxicity of the drug (1). It is estimated that developing a single drug candidate costs around 824 million dollars over 5.5 y, including steps of target screening, lead target selection, chemical inhibitor development, and preclinical testing (1, 2). Regardless, about 60% of drug candidates fail at the preclinical stage in large part due to poor drug efficacy and/or intolerable systemic toxicities (1, 3). Unfortunately, such adverse observations can stall drug development in the absence of essential knowledge as to whether the poor preclinical or clinical results are a failure of the target or the drug. Therefore, cost-effective strategies to provide timely information related to efficacy and toxicity of target inhibition would focus resources on the most robust targets.

The pharmaceutical development of CDK9 inhibitors is a representative example of some of these bottlenecks in the drug target validation process. CDK9 is a serine–threonine kinase that forms the catalytic core of positive transcription elongation factor b (P-TEFb) in the presence of cyclin T, driving RNA polymerase II (RNA Pol II) for transcriptional elongation (4). Owing to its crucial function in transcription elongation and the fact that cancer cells demand high transcriptional activity, CDK9 has emerged as a potential drug target for many cancer types (5). For instance, CDK9 has been proposed as an attractive drug target for acute myeloid leukemia, multiple myeloma, chronic lymphocytic leukemia, hepatocellular carcinoma (HCC), prostate cancer, and also several inflammatory diseases (6–12). These observations have motivated the development of at least 15 different CDK9 inhibitors to date (13).

Still, most pan-CDK inhibitors targeting CDK9, such as flavopiridol, have produced undesirable toxicities in preclinical studies and/or clinical trials, including particularly concerning blood toxicities like neutropenia (7). However, as is the case with many other drugs in development, it remains unclear whether the poor therapeutic index is a shortcoming of the drugs or the actual drug target. For example, most CDK9 inhibitors are not specific, making it difficult to distinguish whether observed toxicities are on- or off-target effects (7). Additionally, their toxicities have not been explored in sufficient detail to determine whether they are reversible. More broadly, most preclinical efficacy studies have relied on xenograft models that are immune deficient, thereby ignoring fundamental aspects of the tumor microenvironment and critical immunological antitumor responses in spontaneous cancer models and in patients (14).

To address these issues, we developed a flexible and versatile mouse model system that uses genetic approaches to enable the assessment of systemic on-target toxicities and anticancer efficacy in the same animal. This system combines two modeling approaches: The production of tetracycline (doxycycline [Dox])-regulated short hairpin RNA (shRNA) mice capable of systemic suppression of a target of interest (15) combined with nongermline methods to somatically deliver genetic elements needed to produce genetically defined tumors. Using CDK9 inhibition as a paradigm, we produced models in which *Cdk9* can be systemically suppressed in tumor-bearing mice and compared the results of genetic *Cdk9* inhibition to those achieved using a recently developed highly specific CDK9 inhibitor. Our results identify a physiologically actionable therapeutic window at which CDK9 inhibition is effective, thereby supporting the utility and predictive value of this time- and cost-effective platform to reveal previously inaccessible information (such as on-target reversible toxicities) relevant to the clinical development of candidate cancer targets.

## Results

### *Cdk9* shRNA Transgenic Mice Allow Temporal Control of *Cdk9* Expression throughout Murine Organs and Tissues.

We previously produced germline models harboring Dox-inducible shRNAs capable of inducible suppression of a therapeutic target (15), and, for other purposes, nongermline mouse models were developed in which genetically defined cancers can be produced in various tissues by somatic tissue engineering (16–18). We envisioned that combining these two approaches would produce a generalizable platform in which the effects of target inhibition on tumors and normal tissues could be evaluated in the same animal, approximating the therapeutic index of the target in vivo. As proof of concept, we explored the effects of *Cdk9* inhibition in mice harboring *MYC*-driven HCCs, which can be hypersensitive to *Cdk9* inhibition (6).

**Fig. 1.**
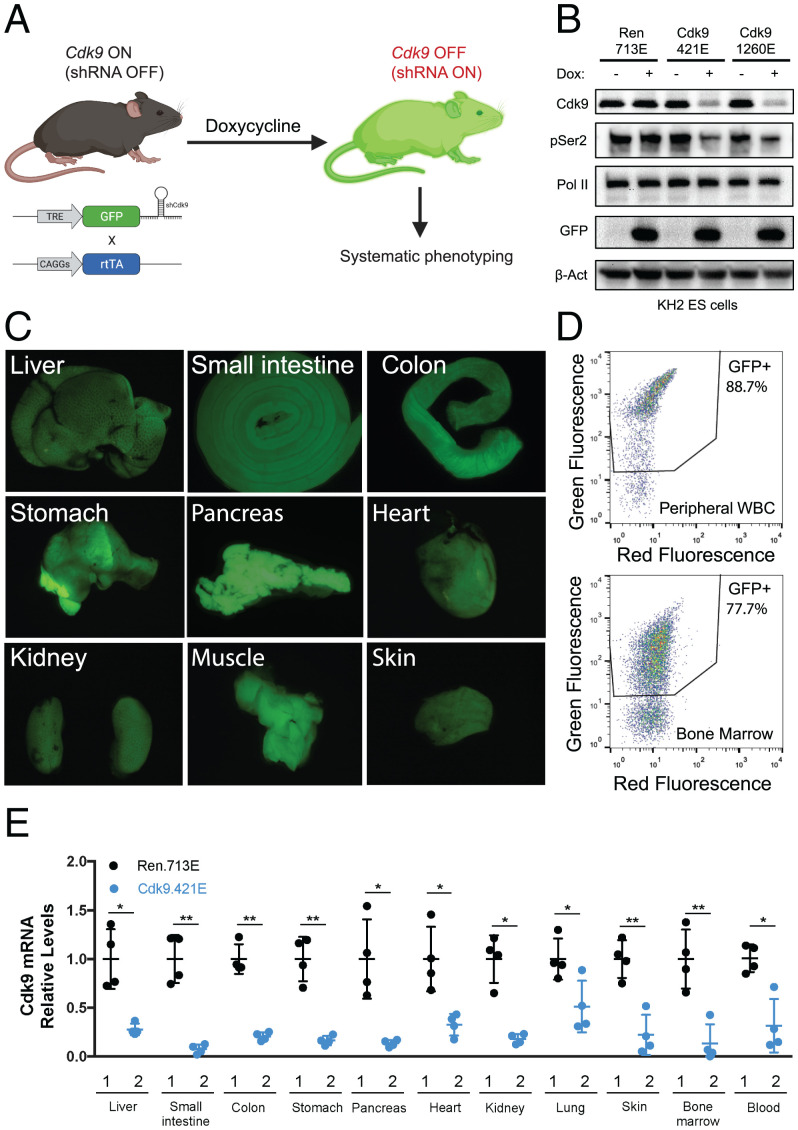
Construction of the transgenic *Cdk9* shRNA mouse model. (*A*) Schematic of plasmid constructs and generation of the CAGs-rtTA3/+; TG-Ren.713E (neutral control), CAGs-rtTA3/+; TG-Cdk9.421E, and CAGs-rtTA3/+; TG-Cdk9.1260E mouse strains. (*B*) Western blot analyses of CDK9 and RNA Pol II pSer2 inhibitions for 60 h of Dox treatment in ES cell clones containing TRE-GFP-miRE Ren.713E or two *Cdk9* miRE shRNAs (Cdk9.421E and Cdk9.1260E). (*C* and *D*) Systemic expression of GFP in organs and tissues and the hematological system from CAGs-rtTA3/+; TRE-GFP-miRE Cdk9.421E mice, maintained on a Dox diet for 72 h. (*E*) Quantitative RT-PCR of *Cdk9* in multiple organs from “1”: CAGs-rtTA3/+; TG-Ren.713E and “2”: CAGs-rtTA3/+; TG-Cdk9.421E mice on a Dox diet for 72 h. Results are demonstrated by four biological replicates and three technical replicates. Statistical significance was calculated by two-tailed Student’s *t* test. Error bars correspond to mean ± SEM (*P < 0.05; **P < 0.01).

The first step in this pipeline involves production of Dox-inducible shRNA transgenic mice. Using a previously optimized approach (15), we integrated Dox-inducible GFP-coupled shRNAs into KH2 embryonic stem (ES) cells harboring a “homing cassette” at the *ColA1* locus, thereby facilitating efficient and reproducible gene targeting using recombination-mediated cassette exchange (Fig. 1*A*). Of note, this also eliminates variable expression of integrated transgenes (“founder effects”) seen using traditional transgenesis approaches (19). To control for sequence-based off-target effects of RNA interference (RNAi), we targeted two distinct but well-validated *Cdk9* shRNAs (Cdk9.421E and Cdk9.1260E) (6, 20). To control for off-target effects associated with shRNA engagement of the RNAi machinery, as well as physiological consequences of Dox, we used a Renilla luciferase–targeting shRNA that efficiently enters the RNAi pathway but has no actual target in normal murine cells (Renilla.713E). As predicted, ES cell clones targeted with two independent *Cdk9* miR-E shRNAs (transgenic [TG]-Cdk9.421E and TG-Cdk9.1260E) showed GFP induction and robust CDK9 depletion when cultured for 60 h in the presence of Dox (Fig. 1*B*). Importantly, *Cdk9* suppression decreased the levels of Ser2 phosphorylation of RNA Pol II (Pol II pSer2, a biomarker of CDK9 activity), indicating that these shRNAs disrupt CDK9 function with high potency (Fig. 1*B*).

We used these ES cell clones to generate chimeric mice via blastocyst injection, and founders were backcrossed to establish germline transmission. Although we have previously demonstrated that shRNA transgenes expressed from the TRE-tight promoter are not leaky, we noticed substantial variability of transgene expression between tissues, with particularly poor expression in the liver (20, 21). To mitigate these issues, we crossed TG-Cdk9 miR-E mice with CAGs-rtTA3 transgenic animals (Fig. 1*A*), which enables stronger and more ubiquitous expression of the TRE-GFP-shRNA cassette (22, 23). As a result, robust GFP expression was observed in the majority of organs harvested from CAGs-rtTA3; TG-shRNA mice (Fig. 1 *C* and *D*) within 72 h of Dox addition, with a corresponding decrease in *Cdk9* expression in these organs and tissues (Fig. 1*E*). Notably, GFP was poorly expressed in the brain, likely due to the poor ability of Dox to cross the blood brain barrier at this dosage with an ad libitum Dox diet (24). Thus, CAGs-rtTA3; TG-Cdk9 mice enable inducible and systemic *Cdk9* inhibition across a multitude of organs and tissues of adult mice, which we hypothesized could be leveraged to assess the validity and toxicity of CDK9 as an anticancer drug target.

### A Transgenic shCdk9 Mouse Model Identifies Reversible Pathologies in Multiple Tissues.

**Fig. 2.**
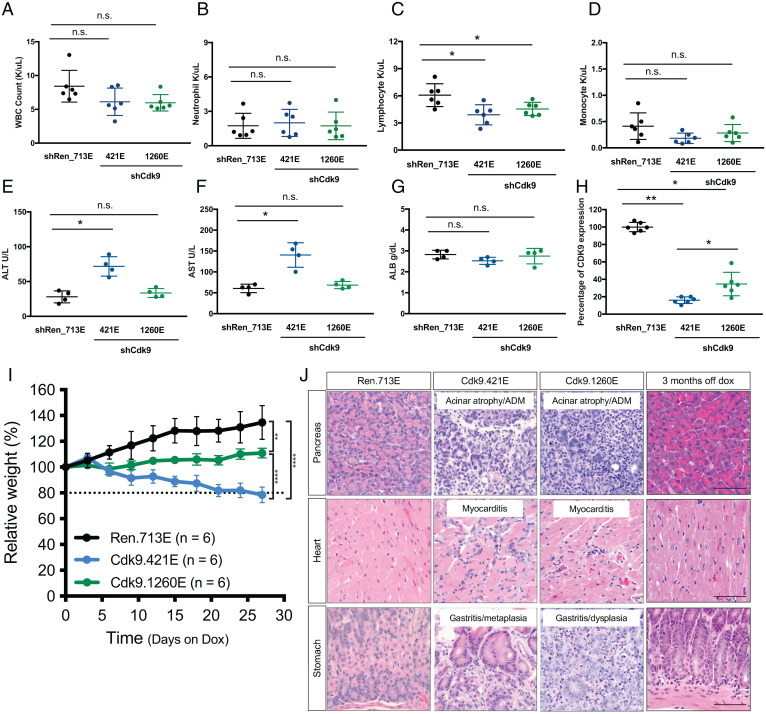
*Cdk9* suppression results in reversible toxicities in selected organs and tissues in the inducible shCdk9 mouse model. (*A*–*D*) Total WBC, neutrophil, lymphocyte, and monocyte counts of CAGs-rtTA3/+; TG-Ren.713E, TG-Cdk9.421E, and TG-Cdk9.1260E mice on a Dox diet for 2 wk. Results are demonstrated by six biological replicates in each genotype of mice. Statistical significance was calculated by two-tailed Student’s *t* test. Error bars correspond to mean ± SEM (*P < 0.05; n.s., not significant). (*E*–*G*) Serological analysis of the concentrations of ALT, AST, ALB in CAGs-rtTA3/+; TG-Ren.713E, TG-Cdk9.421E, and TG-Cdk9.1260E mice on a Dox diet for 2 wk. Results are demonstrated by four biological replicates in each genotype of mice. Statistical significance was calculated by two-tailed Student’s *t* test. Error bars correspond to mean ± SEM (*P < 0.05; **P < 0.01; n.s., not significant). (*H*) Quantification of CDK9 expression based on immunohistochemistry (IHC) staining of tissue sections from CAGs-rtTA3/+; TG-Ren.713E, TG-Cdk9.421E, and TG-Cdk9.1260E mice on a Dox diet for 2 wk. Results are demonstrated by three biological replicates and two technical replicates in each genotype of mice. Statistical significance was calculated by two-tailed Student’s *t* test. Error bars correspond to mean ± SEM (*P < 0.05; **P < 0.01). (*I*) Weight changes of CAGs-rtTA3/+; TG-Ren.713E, TG-Cdk9.421E, and TG-Cdk9.1260E mice on the Dox diet, relative to Day 0 of the Dox diet treatment. Results are demonstrated by six biological replicates in each genotype of mice. Statistical significance was calculated by two-tailed Student’s *t* test. Error bars correspond to mean ± SEM (**P < 0.01; ****P < 0.0001). (*J*) Representative hematoxylin and eosin (H&E) staining images of pancreas, heart, and stomach sections from CAG-rtTA3/+; TG-Ren.713E (4 wk on the Dox diet), TG-Cdk9.421E (4 wk on the Dox diet), TG-Cdk9.1260E (4 mo on the Dox diet and 3 mo post-Dox withdrawal, respectively). (Scale bar, 50 μm.)

The various nonspecific CDK9 inhibitors that have been tested in preclinical and clinical settings produce a range of toxicities and side effects that affect the stomach and liver and can also trigger hypokalemia and neutropenia (7, 25). To examine the specific consequences of CDK9 protein suppression, we treated adult CAGs-rtTA; TG-Cdk9 (Cdk9.421E and Cdk9.1260E) and CAGs-rtTA3; TG-Renilla (Ren.713E) transgenic mice with Dox and examined their blood and liver function over time. CAGs-rtTA3; TG-Cdk9 mice fed a Dox diet for 2 wk showed a nonstatistically significant decrease of white blood cell (WBC) counts relative to control animals (CAGs-rtTA; TG-Renilla mice) (Fig. 2*A*). Similarly, while neutrophil populations remained unchanged, monocyte and lymphocyte counts trended downward following *Cdk9* inhibition, the latter mostly due to a decrease in B cells (Fig. 2 *B*–*D* and *SI Appendix*, Fig. S1*A*). Serum levels of various proteins indicative of liver damage and/or function, including alanine aminotransferase (ALT), aspartate aminotransferase (AST), and albumin (ALB), remained in the normal range following Dox administration to CAGs-rtTA3; TG-Cdk9.1260E mice and controls (Fig. 2 *E*–*G*). While both AST and ALT levels increased following Dox administration in TG-Cdk9.421E mice, they remained within the healthy range. Of note, the Cdk9.421E shRNA is more potent at suppressing *Cdk9* mRNA compared to TG-Cdk9.1260 (Fig. 2*H*), raising the possibility that its more pronounced effect on liver enzymes reflects a potentially reversible dosage effect.

To gain a more comprehensive understanding of potential toxicities associated with *Cdk9* suppression, we examined a cohort of CAGs-rtTA3; TG-Cdk9.421E, CAGs-rtTA3; TG-Cdk9.1260E, and CAGs-rtTA3; TG-Ren.713E mice fed a Dox diet (Fig. 2 *I* and *J*). Relative to control CAGs-rtTA3; TG-Ren.713E mice, Dox-fed CAGs-rtTA3; TG-Cdk9.1260E mice showed reduced weight gain while CAGs-rtTA3; TG-Cdk9.421E mice showed significant weight loss over 4 wk (Fig. 2*I*). The rate of weight change in CAGs-rtTA3; TG-Cdk9.421E and CAGs-rtTA; TG-Cdk9.1260E mice also directly correlated with the potency of *Cdk9* suppression, with CAGs-rtTA; TG-Cdk9.421E having the most potent activity.

To explore the systemic effects of *Cdk9* suppression in more detail, we performed a comprehensive assessment of all shRNA-expressing/GFP-positive organs throughout the body of CAGs-rtTA3; TG-Cdk9 (Cdk9.421E and Cdk9.1260E) mice in a blinded manner with a trained pathologist. No significant abnormalities were observed after Dox treatment for 2 wk, a frequently used end point in drug toxicology studies. However, moderate to severe pathologies were noted in CAGs-rtTA3; TG-Cdk9.421E mice 4 wk after continuous Dox treatment, including acinar atrophy and acinar-to-ductal metaplasia (ADM) in the pancreas, myocarditis in the heart, and gastritis and metaplasia in the stomach (Fig. 2*J* and *SI Appendix*, Fig. S1*B*). As expected, we observed GFP induction and decreased pSer2 levels in cells and tissues from mice fed with Dox at both short- and long-term time points, which directly correlated with *Cdk9* suppression (*SI Appendix*, Fig. S2 *A*–*D*). Of note, these phenotypes were not observed in CAGs-rtTA3; TG-Cdk9.1260E animals after 4 wk of Dox (*SI Appendix*, Fig. S1*B*) and only became detectable in a subset of CAGs-rtTA3; TG-Cdk9.1260E mice after 16 wk (Fig. 2*J* and *SI Appendix*, Fig. S1*B*). While we cannot rule out the possibility that the more pronounced phenotypes associated with CAGs-rtTA; TG-Cdk9.421E mice are off-target effects, these data suggest that the effects of *Cdk9* inhibition on systemic pathologies are dose dependent.

One unique capability provided by our model is the ability to inactivate the shRNA at different times simply by withdrawing Dox from the diet, thereby restoring endogenous protein expression and enabling an assessment of whether any observed phenotypes are reversible (15, 21). To test this for *Cdk9*, we removed Dox from the diet of CAGs-rtTA3; TG-Cdk9.1260E mice that had received Dox for 16 wk, the time point at which organ pathologies become detectable with this *Cdk9* shRNA. In most CAGs-rtTA3; TG-Cdk9.1260E animals analyzed, tissues from organs affected by *Cdk9* inhibition (heart, stomach, and pancreas) appeared normal after 12 wk of *Cdk9* restoration, while the remaining mouse tissues (∼33.3%) retained detectable pathologies that were substantially reduced compared to the prewithdrawal group (Fig. 2*J* and *SI Appendix*, Fig. S1*B*). These results demonstrate that selective, potent, and sustained *Cdk9* inhibition is not immediately lethal but produces a range of tissue pathologies that show moderate penetrance and are largely reversible.

### Systemic *Cdk9* Suppression in HCC Tumor–Bearing Mice Shows Antitumor Efficacy.

**Fig. 3.**
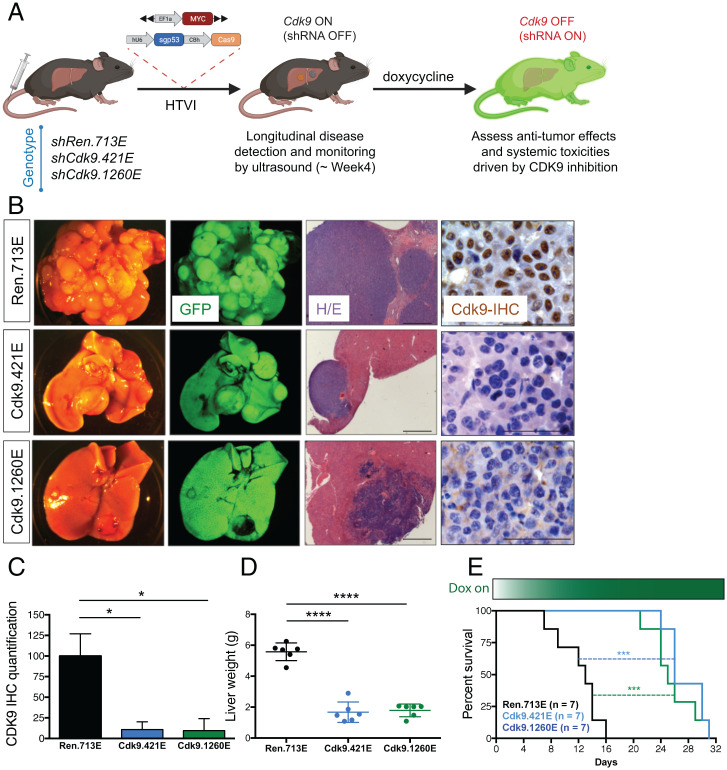
*Cdk9* suppression reduces HCC tumor burden and prolongs survival in a genetically engineered inducible shCdk9 mouse model. (*A*) Diagram depicting generation of autochthonous HCC model in the shRNA transgenic mice using hydrodynamic injection to deliver transposons expressing oncogene *MYC* to the liver together with CRISPR plasmid DNA expressing Cas9 and an sgRNA that directly targets *p53*. (*B*) Representative macroscopic bright-field images, GFP fluorescent images, H&E staining (Scale bar, 500 μm) and CDK9 IHC staining (Scale bar, 50 μm) of liver and tumors from CAG-rtTA3/+; TG-Ren.713E, TG-Cdk9.421E, and TG-Cdk9.1260E mice on the Dox diet for 2 wk. (*C*) CDK9 expression quantification of liver tumors from CAG-rtTA3/+; TG-Ren.713E, TG-Cdk9.421E, and TG-Cdk9.1260E mice on the Dox diet for 2 wk. Results are demonstrated by three biological replicates and three technical replicates in each genotype of mice. Statistical significance was calculated by two-tailed Student’s *t* test. Error bars correspond to mean ± SEM (*P < 0.05). (*D*) Liver weight of mice after the Dox diet for 2 wk upon detection of tumor size within the defined range. Results are demonstrated by six biological replicates in each genotype of mice. Statistical significance was calculated by two-tailed Student’s *t* test. Error bars correspond to mean ± SEM (****P < 0.0001). (*E*) Kaplan–Meier survival curves of tumor-bearing CAG-rtTA3/+; TG-Ren.713E, TG-Cdk9.421E, and TG-Cdk9.1260E mice. Results are demonstrated by seven biological replicates in each genotype of mice. Statistical significance was calculated by Mantel–Cox test (***P < 0.001).

Having established a baseline for the consequences of systemic *Cdk9* inhibition, we set out to investigate the therapeutic index of targeting CDK9 in mice bearing *MYC*-expressing HCCs. We used somatic tissue engineering to produce tumors overexpressing *MYC* and harboring inactivating mutations in *Trp53*, which produces aggressive HCCs in recipient mice without the need for further strain intercrossing (16, 18). Plasmids encoding transposon vectors capable of expressing *MYC*, transposase, and the Cas9 nuclease, together with a constitutive *Trp53*-targeting single guide RNA (sgRNA) (6, 18, 22), were introduced into livers of CAGs-rtTA3; TG-Cdk9 and GAGs-rtTA3; TG-Ren.713E mice using hydrodynamic tail vein injection (HTVI) (Fig. 3*A*) (26), which integrates the *MYC* transgene and disrupts *Trp53* in a subset of hepatocytes. Recipient mice were monitored over time using abdominal palpation and small-animal ultrasound and typically developed aggressive HCCs by 4 to 6 wk (*SI Appendix*, Fig. S3*A*).

To test whether genetic suppression of *Cdk9* impairs tumor maintenance, mice harboring tumors measuring 50 to 100 mm^3^ were fed a Dox diet to systemically induce the *Cdk9* shRNA and control shRNA (Fig. 3 *A* and *B* and *SI Appendix*, Fig. S3*A*). As predicted, and consistent with the observation that most of the liver tumor cells expressed GFP and the *Cdk9* shRNA (Fig. 3*B*), Dox treatment reduced the expression of CDK9 and pSer2 in HCC cells (Fig. 3 *B* and *C* and *SI Appendix*, Fig. S3 *B*–*D*), which correlated with a robust reduction of HCC tumor burden as gauged by liver weight after 2 wk on Dox (Fig. 3*D* and *SI Appendix*, Fig. S3*A*). More importantly, irrespective of the shRNA used, *Cdk9* suppression doubled the median survival, thereby reconfirming the role of CDK9 in HCC maintenance (CAGs-rtTA3; TG-Ren.713E mice: 13 d vs. CAGs-rtTA3; TG-Cdk9.421E and CAGs-rtTA3; TG-Cdk9.1260E mice: 26 and 25 d, respectively) (Fig. 3*E*). The fact that the less potent Cdk9.1260E shRNA was just as effective as the Cdk9.421E shRNA in producing an antitumor outcome yet produces less toxicity is consistent with the hypersensitivity of *MYC*-expressing HCC cells to *Cdk9* inhibition (6). In fact, gene set enrichment analysis (GSEA) of RNA-sequencing (RNA-seq) data from isolated *MYC*-expressing *Cdk9*-suppressed tumor cells (but not controls) showed reversal of signatures associated with MYC expression and ribosome biogenesis (*SI Appendix*, Fig. S3*E*) as was observed in orthotopic models (6, 27). These results establish the feasibility of combining genetically engineered mouse model (GEMM) (shRNA transgenic model) and somatic genome engineering to interrogate factors that influence the therapeutic index of target inhibition.

### High-Dosage Pharmacological Suppression of CDK9 Recapitulates Reversible Tissue Toxicities Predicted by shCdk9 Mice.

Due to the poor selectivity of first-generation pan-CDK inhibitors of CDK9 (13, 28, 29), a new generation of more specific CDK9 inhibitors has been produced (13, 29–31). One such molecule is NVP-2 (30), which inhibits the CDK9-CycT1 catalytic activity and Ser2 phosphorylation of RNA Pol II with significant potency (half maximal inhibitory concentration [IC_50_] values of 0.0005 µM and 0.004 µM, respectively) and specificity (at least 1,200-fold lower activity toward CDK1, 2, 4, and 7) (30). Therefore, to evaluate the predictive power of our modeling approach, we set out to compare the effects of genetic and pharmacologic inhibition of CDK9 on normal tissues and in tumors.

We first examined the effects of NVP-2 on CDK9 activity and gene expression in *MYC;sgp53* murine HCC cell lines (*SI Appendix*, Fig. S4*A*). As expected, pharmacological inhibition of CDK9 using 100 nM NVP-2 led to a decrease in Ser2 phosphorylation in a murine *MYC;sgp53* HCC line, validating that it has on-target activity in our model (*SI Appendix*, Fig. S4*B*). NVP-2 also had a range of effects on the proliferation of a series of human HCC cell lines (*SI Appendix*, Fig. S4 *C*–*F*).

**Fig. 4.**
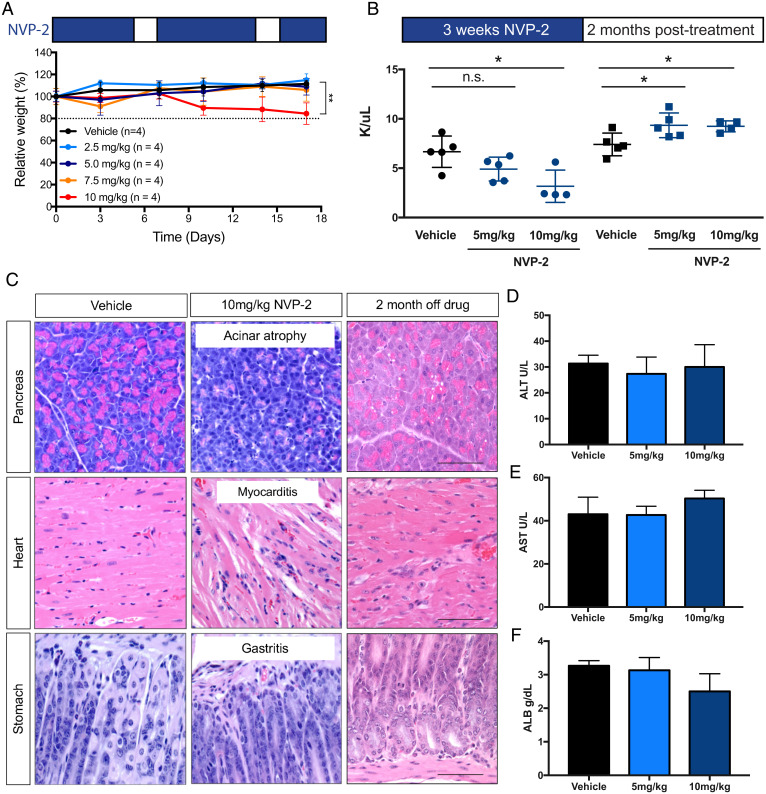
Pharmacological CDK9 suppression results in reversible toxicities in selected organs and tissues. (*A*) Weight changes of wild-type mice treated with vehicle and NVP-2 (2.5 mg/kg, 5 mg/kg, 7.5 mg/kg, 10 mg/kg). Results are demonstrated by four biological replicates in each treatment group. Error bars correspond to mean ± SEM (**P < 0.01). (*B*) Total WBC counts of wild-type mice treated with vehicle and NVP-2 (5 mg/kg, 10 mg/kg) for 3 wk and upon drug withdrawal for 2 mo. Results are demonstrated by four biological replicates in the NVP-2 10 mg/kg group and five biological replicates in the vehicle and NVP-2 5 mg/kg groups. Statistical significance was calculated by two-tailed Student’s *t* test. Error bars correspond to mean ± SEM (*P < 0.05; n.s., not significant). (*C*) Representative H&E staining images of pancreas, heart, and stomach tissue sections from mice with vehicle or NVP-2 treatment (10 mg/kg) for 4 wk and 2 mo upon drug withdrawal. (Scale bar, 50 μm.) (*D*–*F*) Serological analysis of the concentrations of ALT, AST, and ALB in wild-type mice treated with vehicle and NVP-2 (5 mg/kg, 10 mg/kg) for 2 wk. Results are demonstrated by three biological replicates in each group of mice. Statistical significance was calculated by two-tailed Student’s *t* test. Error bars correspond to mean ± SEM (not significant).

To establish a baseline for the effects of pharmacologic CDK9 inhibition in nontumor-bearing animals, wild-type C57BL/6 mice were treated with different doses of NVP-2 (delivered daily, 5 d/wk) and its effects on body weight, blood content, tissue pathology, and liver function were determined. NVP-2 treatment had no effect on body weight in wild-type mice at doses up to 7.5 mg/kg (Fig. 4*A*). Additionally, no effects on blood counts, liver enzymes, and tissue pathology were observed in wild-type mice upon treatment with 5.0 mg/kg of NVP-2 (Fig. 4 *B*–*F*). However, at 10 mg/kg, wild-type animals showed weight loss similar to that produced by the most potent *Cdk9* shRNA (Fig. 4*A*). As has been observed with other CDK9 inhibitors (13), treatment with 10 mg/kg of NVP-2 produced a reduction in WBC counts, including neutrophils (13, 25), which was detectable after 3 wk (Fig. 4*B*). However, these effects were less pronounced than those observed using therapeutically relevant doses of flavopiridol, a potent but nonselective CDK9 inhibitor (32–36), which produced more prominent hematological toxicities (*SI Appendix*, Fig. S5 *A*–*D*). By 4 wk of treatment, the 10 mg/kg NVP-2 dose also produced pathologies in the pancreas, heart, and stomach that were similar to those produced by sustained and prolonged genetic *Cdk9* inhibition (Fig. 4*C*). Nonetheless, but of relevance to our model, serum concentrations of proteins and enzymes linked to liver damage and function were within the normal range at all doses tested (Fig. 4 *D*–*F*).

As was observed with genetic *Cdk9* inhibition, the pathological effects associated with high-dose NVP-2 drug treatment were largely reversible. In fact, animals rebounded with even higher WBC counts than controls by 8 wk after drug withdrawal (Fig. 4*B*). Most of the tissue pathologies observed following 4 wk of 10 mg/kg drug treatment were absent within 8 wk following drug withdrawal, except for cases with moderate lesions in the heart, which were also predicted by our transgenic mouse model (Fig. 4*C*). The fact that systemic and reversible toxicities produced by a selective CDK9 inhibitor were almost identical to those observed in our *Cdk9* GEMM models further underscores the predictive value of this platform.

### Nontoxic Doses of NVP-2 Have Antitumor Activity.

**Fig. 5.**
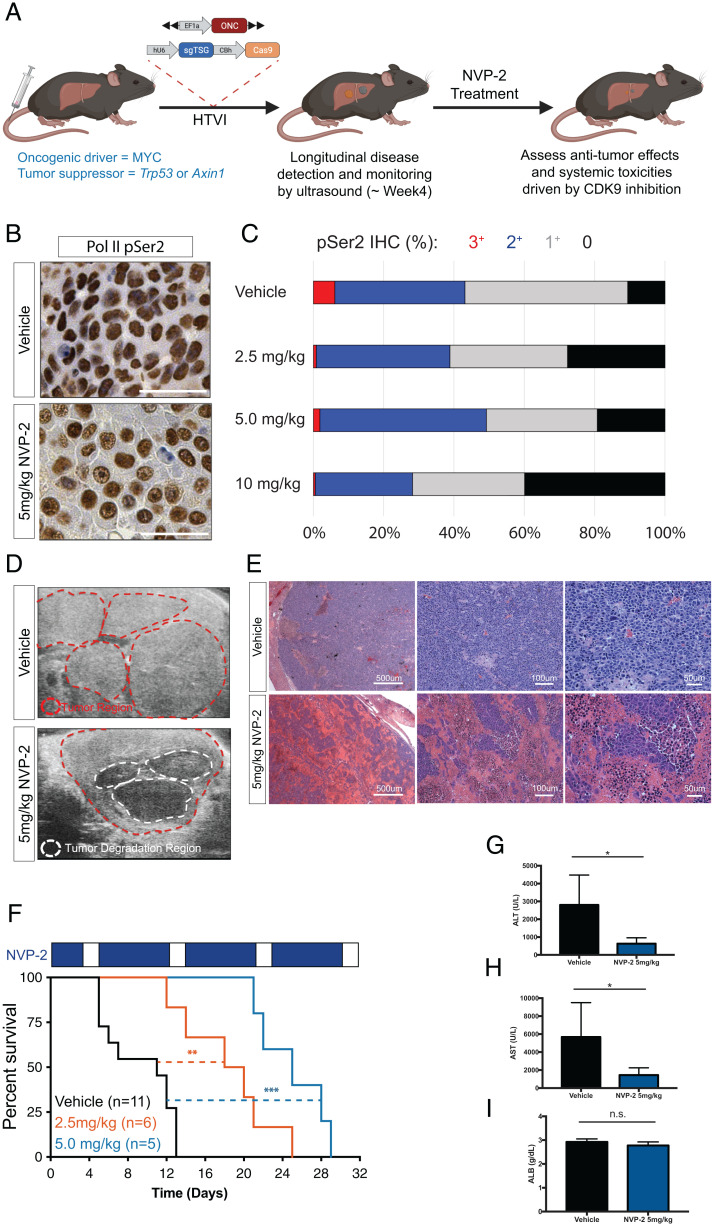
Pharmacological inhibition of CDK9 reduces tumor burden and prolongs survival in HCC mouse model. (*A*) Diagram depicting generation of autochthonous HCC model in wild-type mice using hydrodynamic injection to deliver transposons expressing oncogene *MYC* to the liver together with CRISPR plasmid DNA expressing Cas9 and an sgRNA that directly targets *p53* or *Axin1* (ONC, oncogene; TSG, tumor suppressor gene). (*B* and *C*) Representative RNA Pol II pSer2 IHC staining images and quantification of pSer2 expression in liver tumors with NVP-2 treatment. (Scale bars, 50 μm.) (*D*) Representative ultrasound images of liver and tumor of mice upon NVP-2 or vehicle treatment for 3 wk. Red dotted lines indicate the tumor regions. White dotted lines indicate tumor degradation regions. (*E*) Representative H&E staining images of liver and tumor regions of mice treated with vehicle or NVP-2 for 2 wk. (*F*) Kaplan–Meier survival curves of mice treated with NVP-2 (2.5 mg/kg and 5 mg/kg) and vehicle. Results are demonstrated by indicated number of biological replicates in each group of mice in the graph. Statistical significance was calculated by Mantel–Cox test (**P < 0.01; ***P < 0.001). (*G*–*I*) Serological analysis of the concentrations of ALT, AST, and ALB in HCC-bearing mice on vehicle or NVP-2 (5 mg/kg) treatment for 3 wk. Results are demonstrated by four biological replicates in each group of mice. Statistical significance was calculated by two-tailed Student’s *t* test. Error bars correspond to mean ± SEM (*P < 0.05; n.s., not significant).

To evaluate the therapeutic index of CDK9 inhibition by NVP-2, we tested the antitumor effects of nontoxic doses of NVP-2 in murine liver cancer models driven by *MYC* overexpression and *Trp53* deletion—the same genetic configuration used in our *Cdk9* GEMM studies (Fig. 5*A*). As described above, tumors were produced in wild-type animals using HTVI. Upon tumor manifestation, we treated mice with NVP-2 (2.5 or 5 mg/kg, once per day, 5 d per week) or vehicle followed by an assessment of CDK9 inhibition (pSer2), tumor response (ultrasound, liver enzymes, and histology), and overall survival.

These analyses indicated that the effects of NVP-2 were comparable to genetic suppression of *Cdk9* (*SI Appendix*, Fig. S3 *B* and *C*). Hence, NVP-2 treatment led to decreased Ser2 phosphorylation in tumor cells (Fig. 5 *B* and *C*) and a robust antitumor response associated with tumor regression and massive tumor cell death (Fig. 5 *D* and *E*). Accordingly, NVP-2 treatment produced a statistically significant dose-dependent survival benefit (median survival of vehicle: 11 d; 2.5 mg/kg: 19 d; 5 mg/kg: 25 d) (Fig. 5*F*). While tumor-bearing mice showed no reduction in WBC counts over the course of the experiment (*SI Appendix*, Fig. S5 *E*–*H*), NVP-2 treatment substantially reduced serum levels of ALT and AST compared to the aberrantly high values observed in controls (Fig. 5 *G*–*I*). Of note, while NVP-2–treated mice eventually succumbed to disease, the therapeutic effects were more substantial than those produced by the maximum-tolerated dose of sorafenib, the FDA-approved standard treatment for patients with HCC (*SI Appendix*, Fig. S5 *I* and *J*).

To test whether the observed NVP-2 antitumor effects translated to HCC tumors harboring other genetic lesions, we performed similar treatment studies in mice harboring HCC tumors driven by *MYC* overexpression and *Axin1* deletion, another common event in human HCCs that also drives tumorigenesis in the HTVI system (37). As shown in *SI Appendix*, Fig. S5 *K* and *L*, treatment of tumor-bearing *MYC;sgAxin1* mice with NVP-2 led to statistically significant antitumor responses and an overall extension in median survival from 18.5 d to 40 d. These results demonstrate that the effects of NVP-2 are not limited to a single cancer genotype and further highlight the flexibility of our somatic engineering strategies to test different therapeutic hypotheses in vivo.

## Discussion

Our study develops and credentials a platform for rapid and cost-effective validation and optimization of anticancer drug candidates. It combines the power of GEMMs, somatic genome editing, and inducible-reversible RNAi technologies to evaluate the consequences of target inhibition in tumor and normal cells of the same animal, identifying factors that dictate the therapeutic index of on-target inhibition. As shown here for *Cdk9*, it involves the efficient production of transgenic strains harboring an inducible shRNA capable of suppressing the therapeutic target followed by somatic delivery of elements needed to produce autochthonous genetically defined cancers. The approach is highly modular and could be applied to any drug target with any cancer-associated genotype simply by exchanging the targeted shRNA or incorporating different constructs during somatic tumor initiation experiments.

The system worked well in that while in some settings selection against a deleterious shRNA can lead to accumulation of cells incapable of suppressing the protein over time, we did not observe substantial numbers of cells that escaped *Cdk9* knockdown in the tumors or normal tissues from our mice. Nonetheless, we have noted rare escaping cells in other contexts, so it is important to assess the presence of the linked GFP reporter to confirm whether cells that escape target knockdown are masking important phenotypes. Additionally, while the somatic engineering method used herein is specific to hepatocyte transduction, newer tissue electroporation methodologies developed by our group and others enable the rapid somatic induction of genetically defined cancers in a broad range of tissues and organs, including pancreas and prostate (17, 38). We anticipate that these and other somatic tissue engineering techniques together with our shRNA transgenic approach will open the door for broader systematic drug target validation efforts across a wide range of genotypes and tumor types.

Most efforts focused on genetic validation of therapeutic targets rely on target inhibition via gene manipulation of human-derived cancer cell lines that are subsequently studied as xenografts. In GEMMs, elaborate engineering and intercrossing strategies have been employed to eliminate target function by conditional deletion in the organ of interest (14). While useful, both approaches assess whether a particular gene is required for tumor engraftment or initiation, but not tumor maintenance, and provide no insights into the potential deleterious effects of target inhibition in normal tissues. While the use of constitutive knockout animals can address this in principle, genes encoding many well-validated therapeutic targets are needed for embryonic development, and so studies in adult animals are often impossible. Our approach addresses these issues by allowing spatiotemporal inducible inhibition of the target in both tumor and normal cells of adult animals after tumor formation. Control strains enable simple assessment of inadvertent off-target or Dox effects. In principle, our approach could also be applied to develop rat models, which are commonly used in the drug development industry (39).

Despite known differences between RNAi-mediated protein knockdown and small molecule protein inhibition, we observed remarkably similar phenotypes between shRNA-mediated inhibition of *Cdk9* to those produced by treatment with NVP-2 (30). Accordingly, our platform correctly identified known side effects of CDK9 inhibition in the hematopoietic compartment and noted pathologies in multiple organ systems that were recapitulated in animals following prolonged treatment with NVP-2. As observed in our model, treatment of patients with nonspecific CDK9 inhibitors (e.g., flavopiridol, P276-00, and roscovitine) can cause gastrointestinal mucositis, cardiac complications, weight loss, and mild lymphopenia (32–36, 40–42). Notably, other toxicities, including respiratory disorders, liver dysfunction, and severe hematologic toxicities also observed in patients were not observed in mice following *Cdk9* suppression or following treatment with NVP-2. While it is not possible to precisely match and compare the severity of the toxicities observed in different clinical trials or using agents of different potency, these observations and our results are consistent with the latter toxicities being off-target effects.

The striking concordance between genetic and pharmacological target inhibition may not extend to all therapeutic targets. For example, in some settings, reduction in protein levels may not precisely resemble the consequences of small molecule inhibition, particularly when a target also contains an important scaffolding function that may be left unaffected by the drug. Nevertheless, even in these settings, information on protein loss of function will still provide important clues about target function in a whole animal context, thereby providing significantly more insight relative to analogous genetic studies in cultured cells or xenografts. As protein degradation therapeutics are becoming more common (43), protein knockdown using inducible shRNAs or CRISPR interference are an ideal modality for establishing phenotypes linked to on-target inhibition.

Beyond establishing our modeling approach, our results provide information relevant to the clinical development of CDK9 inhibitors for cancer. First, they establish that on-target inhibition of CDK9 can have potent antitumor effects against genetically defined autochthonous liver cancers in vivo, leading to improved liver function and prolonged survival. Second, they reveal that on-target CDK9 inhibition can produce concerning (but often reversible) toxicities in a subset of animals. Comparing the collective results obtained using different *Cdk9* shRNAs and doses of NVP-2 revealed a clear dose dependence on phenotypes and allowed us to identify a CDK9-targeting regimen that maintains efficacy against tumors without any appreciable toxicity. While it remains to be determined whether similar results can be achieved in cancer patients, a growing number of reports have established CDK9 as a promising target in various cancers and other diseases (6–12). Our results add granularity to on-target phenotypes of CDK9 inhibitors and motivate future studies aimed at leveraging this therapeutic window for anticancer treatments.

**Fig. 6.**
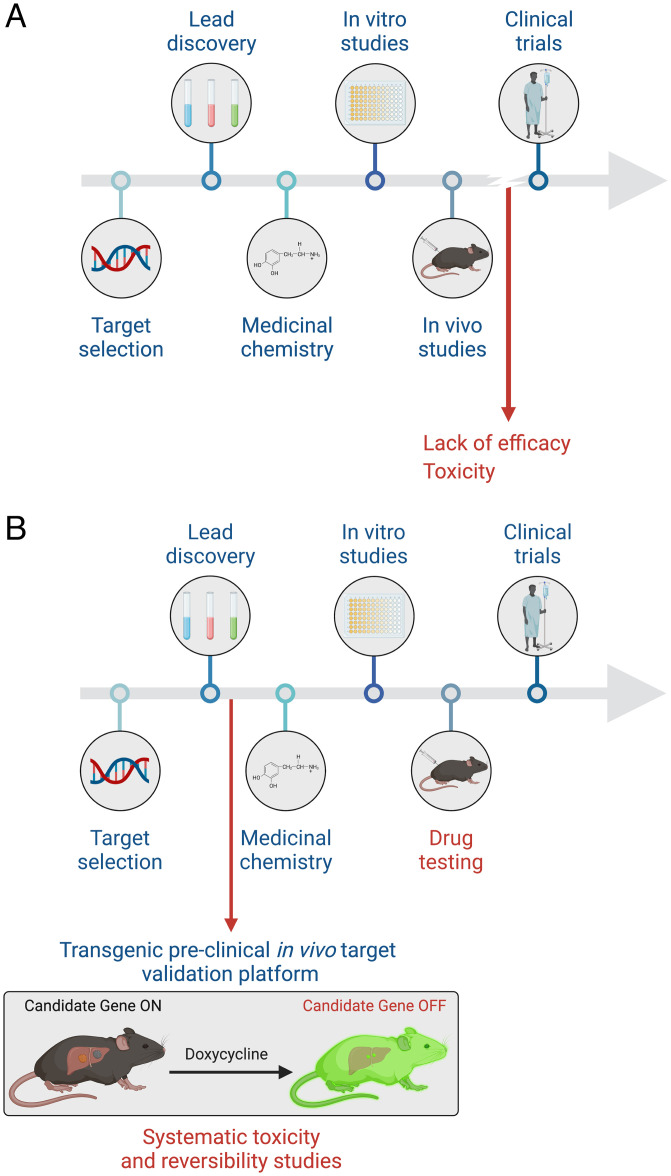
All-in-one mouse model facilitates target validation in drug development. (*A*) Diagram depicting the common current pipeline of drug development. (*B*) All-in-one model as a proposed step of target validation in the drug development pipeline.

In summary, we anticipate that our “all-in-one” platform and variations thereof will be useful for assessing the therapeutic index of candidate drug targets and provide a powerful tool in the drug validation process that can be implemented in parallel with, or even before development and testing of a candidate drug compound(s) (Fig. 6). Given its generalizable and scalable nature, the platform could be leveraged to systematically assess on-target activities and toxicities associated with any current and future candidate small molecules, thereby increasing the opportunity for advancing clinically successful medications.

## Materials and Methods

### Production of Doxycycline-Inducible shRNA Transgenic Mice.

GFP-coupled shRNA constructs (shRen.713E, shCdk9.421E, shCdk9.1260E, respectively) were incorporated into KH2 ES cells harboring a homing cassette at the *ColA1* locus using previously optimized methods (15, 19, 44). The 22-mer guide sequences of shRNAs: shRen.713E (TAGATAAGCATTATAATTCCTA), shCdk9.421E (TCAAACACCAGATAGATGCTGC), and shCdk9.1260E (TAACCTAAGAACAACACCGGCC). Briefly, electroporation of 50 μg pCol-TGM (TRE-GFP-miRE) was performed using two pulses in a Gene Pulser II (Bio-Rad). At 36 to 48 h postelectroporation, GFP was measured by flow cytometry (Guava EasyCyte) to assess the electroporation efficiency. Only cultures with >10% GFP expression were maintained and selected in 140 μg/mL hygromycin.

The selected ES cell clones were used to generate chimeric mice via blastocyst injection using ES cells rederived from E3.5 blastocysts (22, 44). Founders were backcrossed to establish germline transmission. TG-Cdk9 miR-E mice were crossed with CAGs-rtTA3 transgenic animals and genotyped by genomic PCR using shRNA-specific forward primers and common reverse primers: CAGs-rtTA3/+ forward primer: CTGCTGTCCATTCCTTATTC (transgene), TGCCTATCATGTTGTCAAA (wild-type) and reverse primer: CGAAACTCTG GTTGACATG; TG-Ren.713E forward primer: GTATAGATAAGCATTATAATTCCTA and reverse primer: CACCCTGAAAACTTTGCCCC; TG-Cdk9.421E forward primer: TGTATCAAACAC CAGATAGATGC and reverse primer: CACCCTGAAAACTTTGCCCC; and TG-Cdk9.1260E forward primer: TGTATAACCTAAGAACAACACCG and reverse primer: CACCCTGAAAACTTTGCCCC.

### Animal Studies.

All mouse experiments were performed according to the protocols approved by the Memorial Sloan Kettering Cancer Center (MSKCC) Animal Care and Use Committee (protocol no. 11-06-011). Mice were maintained under specific pathogen-free conditions, and food and water were provided ad libitum. Doxycycline was administered to mice via food pellets (625 mg/kg) from Harlan Teklad. NVP-2 was administered to wild-type C57BL/6 mice via oral gavage treatment at indicated dosages (2.5 mg/kg, 5 mg/kg, 7.5 mg/kg, or 10 mg/kg) with 0.5% hydroxypropyl methylcellulose (HPMC) and 0.2% Tween-80 as the solvent (vehicle).

### Immunoblotting.

Liver tissues and cell pellets were lysed in Laemmli buffer or protein lysis buffer (200 mM NaCl, 0.2% Nonidet P-40, 50 mM Tris at pH 7.5, 1% Tween-20, protease, and phosphatases inhibitors) using a tissue homogenizer. Equal amounts of protein were separated on 12% sodium dodecyl sulfate–polyacrylamide gels and transferred to polyvinylidene difluoride (PVDF) membranes. The abundance of β-actin was monitored to ensure equal loading. Images were analyzed using AlphaView software (ProteinSimple). Detection in immunoblots was performed using antibodies for CDK9 (Santa Cruz Biotechnology), phopho-Ser2 RNA Pol II (Bethyl Laboratories), RNA Pol II (Bethyl Laboratories), and GFP (Abcam).

### Flow Cytometry.

Single-cell suspensions were prepared from bone marrow or peripheral blood. Red blood cells were lysed with ammonium–chloride–potassium buffer, and the remaining cells were resuspended in phosphate buffered saline (PBS) with 2% fetal bovine serum (FBS). Nonspecific antibody binding was blocked by incubation with 20 μg/mL rat IgG (Sigma-Aldrich) for 15 min, and cells were then incubated with the primary antibodies for 30 min on ice. Stained cells were quantified using a Fortessa analyzer (BD Biosciences). FlowJo software (TreeStar) was used to generate flow cytometry plots.

### Immunohistochemistry and Immunofluorescence Analysis.

Organ samples were fixed in 10% formalin overnight at 4 °C and further subjected to routine histological procedures for embedding in paraffin. Immunohistochemical and immunofluorescence staining was performed following standard protocols. Images were taken on a Zeiss Axio Imager Z2 system. For protein expression quantification, three sections from each animal were scanned and the images were quantified using NIH ImageJ software. A comprehensive assessment based on the histology of organs and tissues of the mice was performed in a blinded manner with a trained pathologist.

### RNA Expression Analysis.

For quantitative RT-PCR, total RNA was isolated using the RNeasy Mini Kit (Qiagen), and cDNA was obtained using TaqMan reverse transcription reagents (Applied Biosystems). Real-time PCR was performed in triplicate using SYBR Green PCR Master Mix (Applied Biosystems) on the ViiA 7 Real-Time PCR System (Invitrogen). *β-Actin* and *Gapdh* served as endogenous normalization controls. Gene-specific primer sets were designed using National Center for Biotechnology Information’s qPrimerDepot (https://www.ncbi.nlm.nih.gov/tools/primer-blast/) (*Cdk9* forward primer: TCATGCAGGGTAACACAGAGC and reverse primer: CTTCTGGCCCTTCACAA GTTC; *β-actin* forward primer: CATGTACGTTGCTATCCAGGC and reverse primer: CTCCTTAA TGTCACGCACGAT; and *Gapdh* forward primer: TGATGACATCAAGAAGGTGGTG and reverse primer: TCCTTGGAGGCCATGTGGGCCA). For high-throughput RNA-seqs, total RNA was extracted using an RNeasy minikit (Qiagen). RNA-seq library construction and sequencing were performed at the integrated genomics operation core at MSKCC according to standard protocols. Poly-A selection was performed. For sequencing, ∼10 million 50-bp paired-end reads were acquired per replicate condition. Resulting RNA-seq data were analyzed by removing adaptor sequences using Trimmomatic (45). RNA-seq reads were then aligned to GRCh37.75 (hg19) with STAR (46) and genome-wide transcript counting was performed by HTSEq (47) to generate a matrix of fragments per kilobase of exon per million fragments mapped.

### Small-Animal Ultrasound Imaging.

For in vivo quantification and imaging of liver tumors, mice were scanned using a FujiFilm-Vevo 2100. Approximately 5 min before recording positron emission tomography (PET) images, mice were anesthetized by inhalation of 1 to 2% isoflurane (Isothesia, Henry Schein Animal Health) in an oxygen gas mixture and then placed on a scanner bed. Image reconstruction and processing details have been previously reported (48).

### Serological Analysis and Blood Cell Count.

Blood samples were collected according to the animal protocol from the submandibular vein of mice. Whole blood sample was used for blood cell (including subpopulations of neutrophils, lymphocytes, and monocytes) analysis with Hemavet (Drew Scientific). Serum samples were prepared after centrifugation for the analysis of the levels of ALT, AST, and ALB using an AU680 Clinical Chemistry Analyzer (Beckman Coulter).

### HTVI.

In preparation for the procedure, the mouse was carefully warmed with a heat lamp to cause vasodilation. The needle was directed into a lateral tail vein at an angle of ∼20°. Once the vein was penetrated (preferably midway down the tail), the needle was directed cranially at a distance of ∼2 mm. Sterile 0.9% NaCl solution containing 5 µg of DNA of pT3-EF1a-Myc and 20 µg of CRISPR plasmid px330 DNA (49) expressing Cas9 and sgRNA targeting either *p53* or *Axin1* together with CMV-SB13 transposase (1:5 ratio) for each injection was administered in 5 to 7 s (18). Pressure was applied over the injection site after the needle was withdrawn from the vein for ∼30 s with gauze (or similar material) (26).

### GSEA.

GSEA (50) was performed using GSEA v2.07 software. A detailed description of GSEA methodology and interpretation is provided at https://www.gsea-msigdb.org/gsea/doc/GSEAUserGuideFrame.html. Significance of gene sets from the GSEA was based on the normalized enrichment score (NES) and the false discovery rate q-value (FDR q-val) to determine the probability that a gene set with a given NES represents a false-positive finding.

### Analysis of the Cancer Cell Line Encyclopedia.

The transcriptome expression profiles of HCC cell lines (SKHep1, HepG2, Alexander, Hep3B, JHH7, and SNU475) were obtained from the Cancer Cell Line Encyclopedia (https://sites.broadinstitute.org/ccle). The Pearson correlation coefficient of the MYC expression level (Log2) (51) and corresponding NVP-2 IC_50_ values (Log10) were calculated using GraphPad Prism software.

### Statistics.

Statistical significance was calculated by two-tailed Student’s *t* test and Mantel–Cox test with GraphPad Prism software. GraphPad Prism software was used to calculate the IC_50_ values. Statistically significant differences are indicated with asterisks in the figures, accompanied by *P* values in the figure legends. Error bars indicate SEM for the number of replicates, as indicated in the figure legends.

## Supplementary Material

Supplementary File

## Data Availability

RNA-seq data have been deposited in Gene Expression Omnibus under accession GSE196783 (https://www.ncbi.nlm.nih.gov/geo/query/acc.cgi?acc=GSE196783).
