# Supplementary material for: A preclinical platform for assessing antitumor effects and systemic toxicities of cancer drug targets
Source: Proc Natl Acad Sci U S A. 2022 Apr 20;119(17):e2110557119. doi: 10.1073/pnas.2110557119 (PMC9169916; doi:10.1073/pnas.2110557119)
Supplement: Supplementary File [file pnas.2110557119.sapp.pdf]

PNAS
www.pnas.org

# Supplementary Information for

# A preclinical platform for assessing anti-tumor effects and systemic toxicities of cancer drug targets

Xiang Li,[1,2,12] Chun-Hao Huang,[1,2,12] Francisco. J. Sánchez-Rivera[1,10,11], Margaret C. Kennedy[1], Darjus F. Tschaharganeh,[1] John P. Morris IV,[1] Antonella Montinaro,[3] Kevin P. O'Rourke,[1,4] Ana Banito,[1] John E. Wilkinson,[5] Chi-Chao Chen,[1,2] Yu-Jui Ho,[1] Lukas E. Dow,[1] Sha Tian,[1] Wei Luan,[1] Elisa de Stanchina,[1] Tinghu Zhang,[6] Nathanael S. Gray,[6] Henning Walczak,[3,7,8] Scott W. Lowe,[1,2,9*]

[1]Cancer Biology and Genetics Program, Memorial Sloan-Kettering Cancer Center, New York, NY 10065

[2]Weill Cornell Graduate School of Medical Sciences, Cornell University, New York, NY 10021

[3]Centre for Cell Death, Cancer, and Inflammation (CCCI), UCL Cancer Institute, University College London, 72 Huntley Street, London WC1E 6DD, UK

[4]Weill Cornell Medicine/The Rockefeller University/Sloan Kettering Institute Tri-Institutional MD-PhD Program, New York, NY 10065

[5]Department of Pathology, University of Michigan School of Medicine, Ann Arbor, MI 48109

[6]Previous address: Dana Farber Cancer Institute, Longwood Center, Room 2209, 360 Longwood Avenue, Boston, MA 02215. Current address: Innovative Medicines Accelerator, Stanford Chemistry, Engineering & Medicine for Human Health (ChEM-H), Stanford University, Stanford, CA 94305

[7]Cellular Stress Responses in Aging-Associated Diseases (CECAD), Cluster of Excellence, University of Cologne, Cologne 50931, Germany

[8]Center for Biochemistry, Medical Faculty, University of Cologne, 50931 Cologne, Germany

[9]Howard Hughes Medical Institute (HHMI), Memorial Sloan Kettering Cancer Center, New York, NY 10065

[10]Current affiliation/address: David H. Koch Institute for Integrative Cancer Research, Massachusetts Institute of Technology, Cambridge, MA 02142

[11]Current affiliation/address: Department of Biology, Massachusetts Institute of Technology, Cambridge, MA 02142

[12]These authors contributed equally.

[*] To whom correspondence may be addressed.

**Corresponding author**

Scott Lowe – lowes@mskcc.org

**This PDF file includes:**

- Figures S1 to S5
- Legends for Figures S1 to S5
- Reference of Supplementary Figures

**Fig. S1.**

**A**

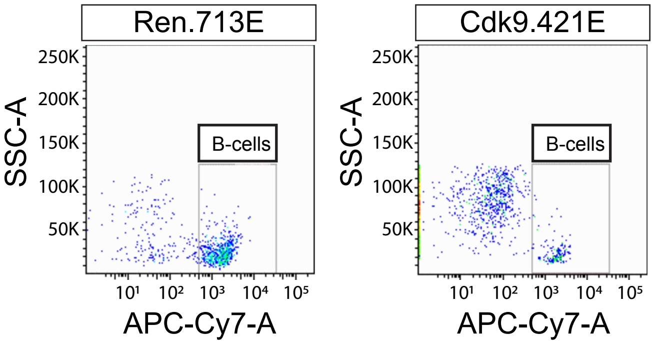


**B**

| Time on Dox Diet | Genotype | Organ/Tissue | Total Number of Animal | Number of Animal with Tissue Lesion | Severity of Lesion | | |
|---|---|---|---|---|---|---|---|
| | | | | | Mild | Moderate | Severe |
| 4 Weeks | CAG-rtTA; TG-Ren.713E mice | Pancreas | 3 | 0 | 0 | 0 | 0 |
| | | Stomach | | 0 | 0 | 0 | 0 |
| | | Heart | | 0 | 0 | 0 | 0 |
| 4 Weeks | CAG-rtTA; TG-Cdk9.421E mice | Pancreas | 4 | 4 | 0 | 2 | 2 |
| | | Stomach | | 4 | 0 | 0 | 4 |
| | | Heart | | 4 | 0 | 0 | 4 |
| 4 Weeks | CAG-rtTA; TG-Cdk9.1260E mice | Pancreas | 4 | 0 | 0 | 0 | 0 |
| | | Stomach | | 0 | 0 | 0 | 0 |
| | | Heart | | 0 | 0 | 0 | 0 |
| 4 Months | CAG-rtTA; TG-Cdk9.1260E mice | Pancreas | 4 | 2 | 0 | 1 | 1 |
| | | Stomach | | 4 | 1 | 2 | 1 |
| | | Heart | | 3 | 2 | 1 | 0 |
| 4 Months on dox<br>3 months off dox | CAG-rtTA; TG-Cdk9.1260E mice | Pancreas | 4 | 1 | 1 | 0 | 0 |
| | | Stomach | | 1 | 0 | 1 | 0 |
| | | Heart | | 2 | 1 | 1 | 0 |

**Supplementary Figure 1. *Cdk9* suppression results in reversible toxicities in selected organs and tissues in the inducible shCdk9 mouse model** (A) Representative flow cytometry analysis of B cell population in the peripheral blood of CAG-rtTA3/+; TG-Ren.713E and CAG-rtTA3/+; TG-Cdk9.421E mice after 2 weeks on Dox diet. (B) Table demonstrating tissue lesion characterization in CAG-rtTA3/+; TG-Ren.713E and CAG-rtTA3/+; TG-Cdk9 mice.

**Fig. S2.**

**A**

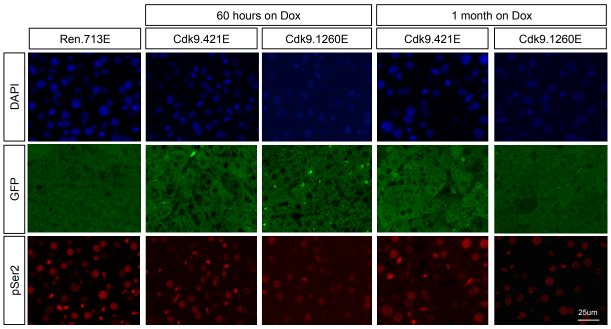

60 hours on Dox
1 month on Dox
Ren.713E
Cdk9.421E
Cdk9.1260E
Cdk9.421E
Cdk9.1260E
DAPI
GFP
pSer2
25um


**B**

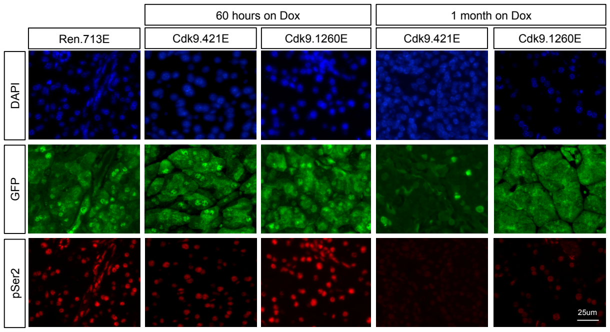

60 hours on Dox
1 month on Dox
Ren.713E
Cdk9.421E
Cdk9.1260E
Cdk9.421E
Cdk9.1260E
DAPI
GFP
pSer2
25um


**C**

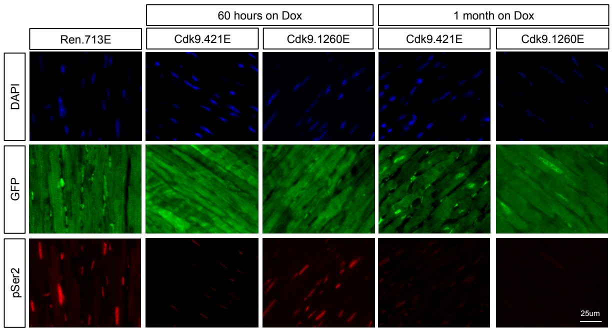

60 hours on Dox
1 month on Dox
Ren.713E
Cdk9.421E
Cdk9.1260E
Cdk9.421E
Cdk9.1260E
DAPI
GFP
pSer2
25um


**D**

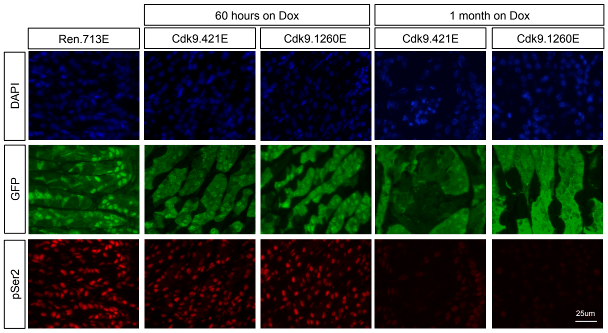

60 hours on Dox
1 month on Dox
Ren.713E
Cdk9.421E
Cdk9.1260E
Cdk9.421E
Cdk9.1260E
DAPI
GFP
pSer2
25um

**Fig. S2. (Continued)**

**Supplementary Figure 2. The effect of *Cdk9* suppression in selected organs and tissues in the inducible shCdk9 mouse model** Representative GFP and RNA Pol II pSer2 immunofluorescence staining images (Scale bars represent 25um.) in the (A) liver, (B) pancreas, (C) heart, and (D) stomach of CAG-rtTA3/+; TG-Ren.713E, TG-Cdk9.421E, and TG-Cdk9.1260E mice upon short-term (60 hours) and long-term (1 months) of Dox treatment.

**Fig. S3.**

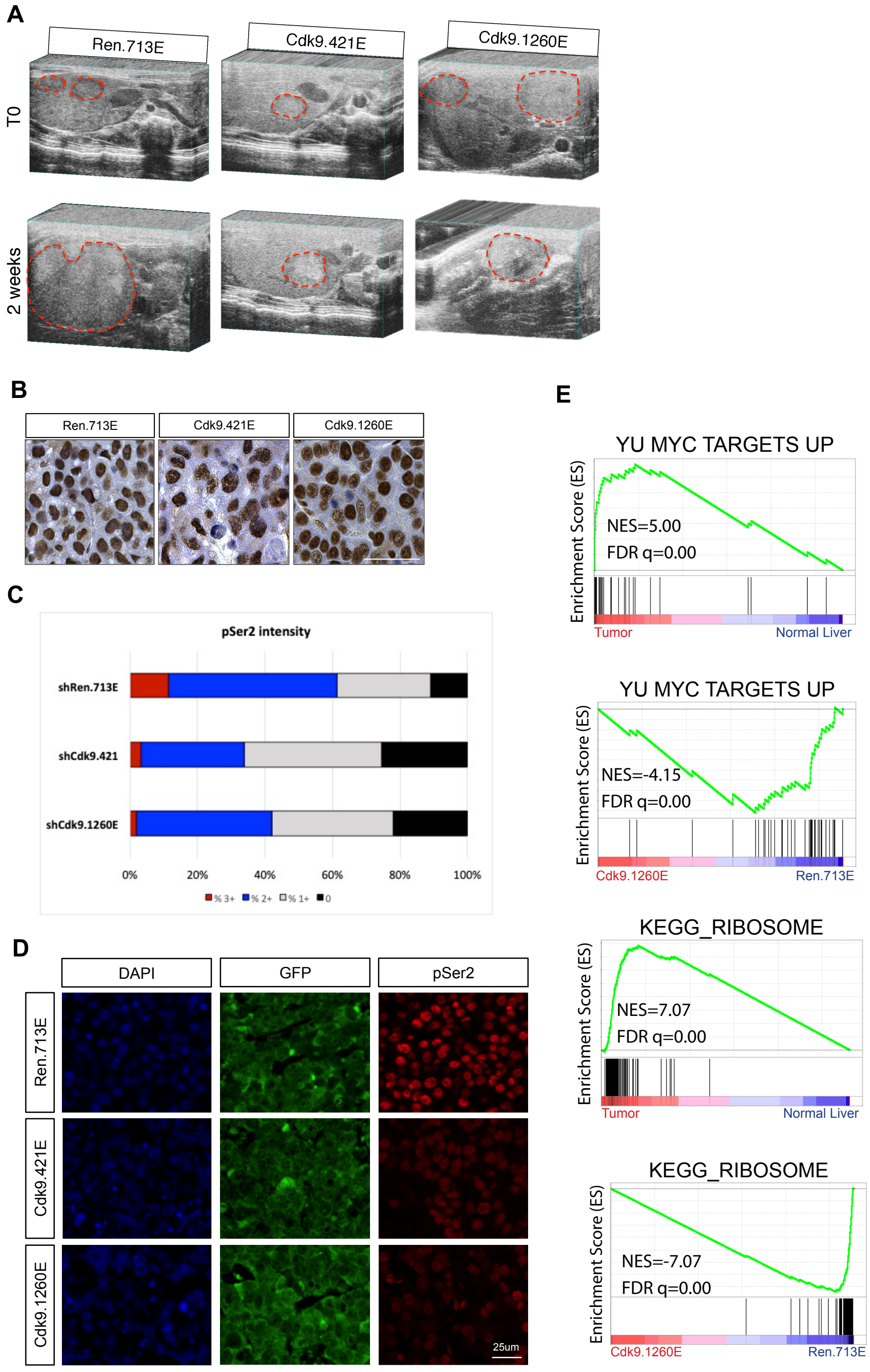
A
Ren.713E
Cdk9.421E
Cdk9.1260E
T0
2 weeks
B
Ren.713E
Cdk9.421E
Cdk9.1260E
C
pSer2 intensity
shRen.713E
shCdk9.421
shCdk9.1260E
0%
20%
40%
60%
80%
100%
% 3+
% 2+
% 1+
0
D
DAPI
GFP
pSer2
Ren.713E
Cdk9.421E
Cdk9.1260E
25um
E
YU MYC TARGETS UP
Enrichment Score (ES)
NES=5.00
FDR q=0.00
Tumor
Normal Liver
YU MYC TARGETS UP
Enrichment Score (ES)
NES=-4.15
FDR q=0.00
Cdk9.1260E
Ren.713E
KEGG_RIBOSOME
Enrichment Score (ES)
NES=7.07
FDR q=0.00
Tumor
Normal Liver
KEGG_RIBOSOME
Enrichment Score (ES)
NES=-7.07
FDR q=0.00
Cdk9.1260E
Ren.713E

**Fig. S3. (Continued)**

**Supplementary Figure 3. *Cdk9* suppression reduces HCC tumor burden and prolongs survival in genetically engineered inducible shCdk9 mouse model**

(A) Representative ultrasound images of livers in CAG-rtTA3/+; TG-Ren.713E, TG-Cdk9.421E and TG-Cdk9.1260E mice at Time 0 (beginning of the experiment) and on the Dox diet for 2 weeks. Red circles indicate areas of liver tumors. (B and C) Representative RNA Pol II pSer2 IHC staining images and quantification of pSer2 expression in liver tumors upon Dox treatment. (Scale bar represents 50um.) (D) Representative GFP and RNA Pol II pSer2 immunofluorescence staining images (Scale bars represent 25um.) in the liver tumors of CAG-rtTA3/+; TG-Ren.713E, TG-Cdk9.421E, and TG-Cdk9.1260E mice upon 2-3 weeks of Dox treatment. (E) GSEA plots evaluating the enrichment of MYC target gene signature and ribosome-related transcriptional signature by comparing *MYC;sgp53* tumors (CAG-rtTA3/+; TG-Ren.713E) versus normal liver and also CAG-rtTA3/+; TG-Cdk9.1260E liver tumors versus CAG-rtTA3/+; TG-Ren.713E liver tumors.

**Fig. S4.**

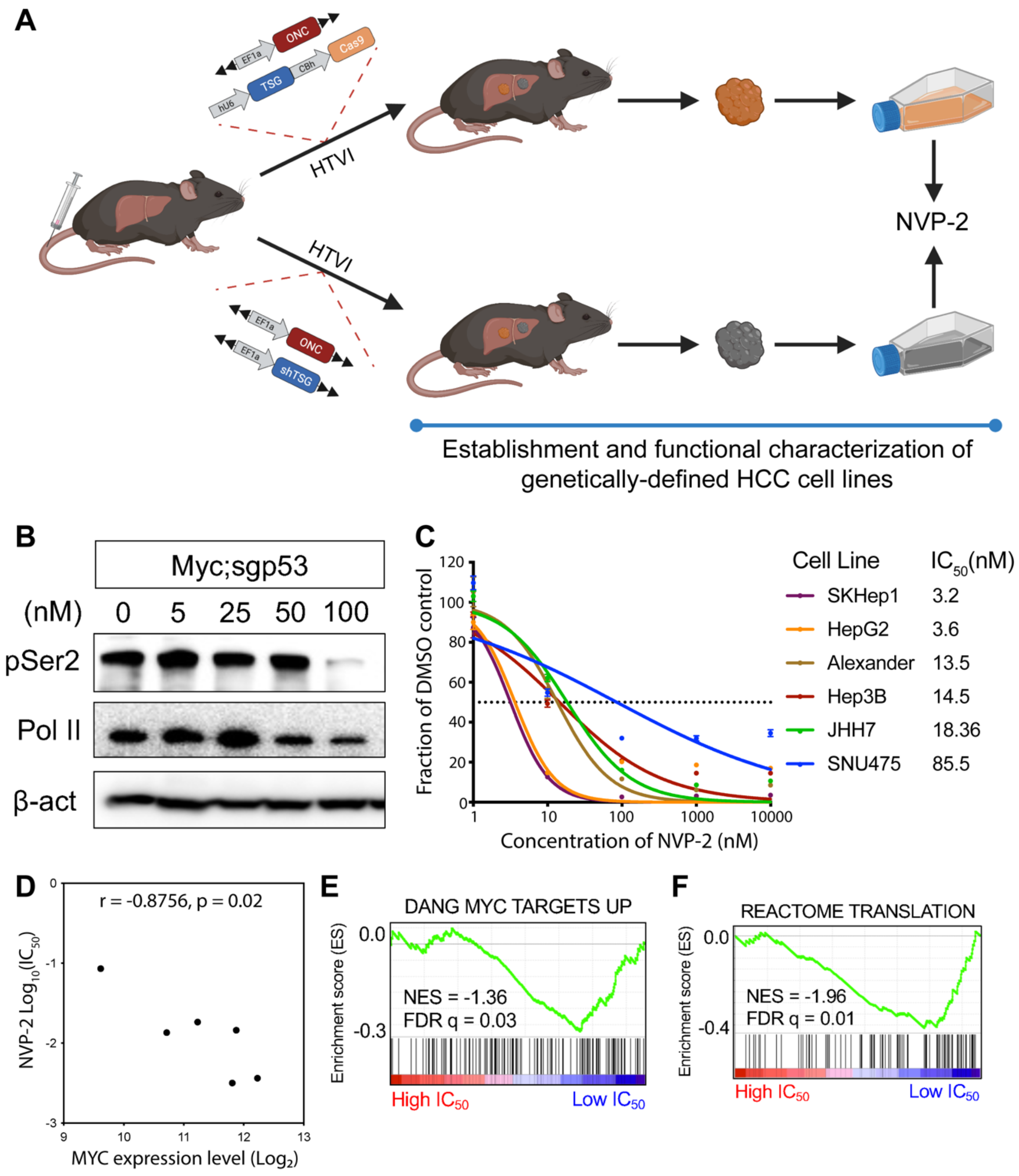
A
ONC
Cas9
EF1a
CBh
TSG
hU6
HTVI
HTVI
NVP-2
EF1a
ONC
EF1a
shTSG
Establishment and functional characterization of genetically-defined HCC cell lines
B
Myc;sgp53
(nM) 0 5 25 50 100
pSer2
Pol II
β-act
C
Fraction of DMSO control
Concentration of NVP-2 (nM)
Cell Line
IC50(nM)
SKHep1 3.2
HepG2 3.6
Alexander 13.5
Hep3B 14.5
JHH7 18.36
SNU475 85.5
D
r = -0.8756, p = 0.02
NVP-2 Log10(IC50)
MYC expression level (Log2)
E
DANG MYC TARGETS UP
Enrichment score (ES)
NES = -1.36
FDR q = 0.03
High IC50
Low IC50
F
REACTOME TRANSLATION
Enrichment score (ES)
NES = -1.96
FDR q = 0.01
High IC50
Low IC50

**Fig. S4. (continued)**

**Supplementary Figure 4. Pharmacological CDK9 suppression results in reversible toxicities in selected organs and tissues** (A) Diagram depicting generation of genetically-defined murine liver cancer cell lines from liver tumors with CRISPR/Cas9 system or potentially with shRNA (ONC: Oncogene; TSG: Tumor suppressor gene). (B) Western blot analysis of RNA Pol II pSer2 level in the *MYC*;sg-*p53* cell line upon NVP-2 treatment. (C) Summary of NVP-2 $IC_{50}$ values based on proliferation rates of NVP-2-treated human HCC cell lines. (D) Scatter plot illustrating the correlation between NVP-2 $IC_{50}$ values and MYC expression levels in the tested human HCC cell lines. (E) GSEA plot evaluating the association between low $IC_{50}$ of NVP-2 and gene signatures of MYC targets (1). (F) GSEA plot evaluating the association between low $IC_{50}$ of NVP-2 and gene signatures of translation (REACTOME).

**Fig. S5.**

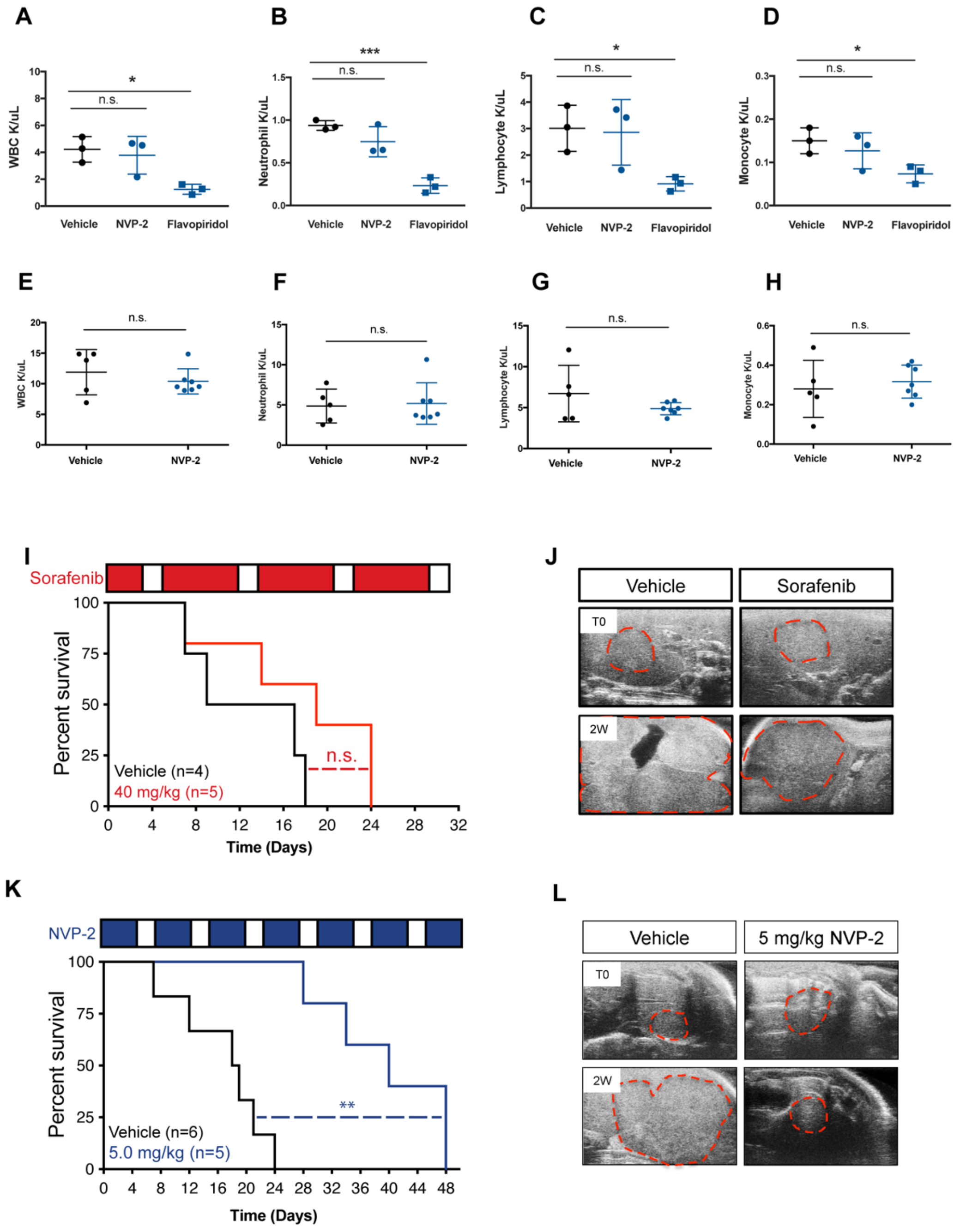

A
WBC K/uL
Vehicle
NVP-2
Flavopiridol
n.s.
*
B
Neutrophil K/uL
***
C
Lymphocyte K/uL
D
Monocyte K/uL
E
F
G
H
I
Sorafenib
Percent survival
Vehicle (n=4)
40 mg/kg (n=5)
Time (Days)
J
Sorafenib
T0
2W
K
Vehicle (n=6)
5.0 mg/kg (n=5)
**
L
5 mg/kg NVP-2

**Fig. S5. (Continued)**

**Supplementary Figure 5. Pharmacological inhibition of CDK9 reduces tumor burden and prolongs survival in HCC mouse model**

(A-D) Total white blood cell, neutrophil, lymphocyte, and monocyte counts in mice on vehicle, NVP-2 (5 mg/kg), or flavopiridol (5mg/kg) treatment for 2 weeks. Results are demonstrated by three biological replicates in each group. Statistical significance was calculated by two-tailed Student's t-test (*, $P < 0.05$; ***, $P < 0.001$). (E-H) Total white blood cell, neutrophil, lymphocyte, and monocyte counts in HCC-bearing mice on vehicle or NVP-2 (5 mg/kg) treatment for 3 weeks. Results are demonstrated by five biological replicates in the vehicle-treated group and seven biological replicates in the NVP-2 treatment group. Statistical significance was calculated by two-tailed Student's t-test. Error bars correspond to mean ± SEM (n.s., not significant). (I) Kaplan-Meier survival curves of HCC (*MYC;sg-p53*)-bearing mice treated with Sorafenib (40 mg/kg) and vehicle. Results are demonstrated by the numbers of biological replicates indicated in the graph. Statistical significance was calculated by Mantel-Cox test (n.s., not significant). (J) Representative ultrasound images of liver and tumor regions of mice in (I) treated with vehicle or sorafenib for 2 weeks. Red circles indicate areas of liver tumors. (K) Kaplan-Meier survival curves of HCC (*MYC;sg-Axin1*)-bearing mice treated with NVP-2 (5 mg/kg) and vehicle. Results are demonstrated by the numbers of biological replicates indicated in the graph. Statistical significance was calculated by Mantel-Cox test (**, $P < 0.01$). (L) Representative ultrasound images of liver and tumor regions of mice in (K) treated with vehicle or NVP-2 for 2 weeks. Red circles indicate areas of liver tumors.

**Reference**

1 Zeller, K. I. et al., An integrated database of genes responsive to the Myc oncogenic transcription factor: identification of direct genomic targets. Genome Biol 4, R69, doi:10.1186/gb-2003-4-10-r69 (2003).
